# Changes in patient-reported outcomes (PROs) and fecal calprotectin levels in patients with inflammatory bowel disease following a three-week gastrointestinal inpatient rehabilitation

**DOI:** 10.1371/journal.pone.0330922

**Published:** 2025-08-29

**Authors:** Marina Kuzdas-Sallaberger, Benedikt Steininger, Doreen Stöhr, Monika Mustak-Blagusz, Christoph Mauel

**Affiliations:** 1 Department for Applied Research, Innovation and Medical Service Development, Pensionsversicherung, Vienna, Austria; 2 Chief Medical Department, Pensionsversicherung, Vienna, Austria; 3 Rehabilitation Center Bad Aussee, Pensionsversicherung, Bad Aussee, Austria; Instituto Nacional de Ciencias Medicas y Nutricion Salvador Zubiran, MEXICO

## Abstract

**Background:**

Inflammatory bowel diseases (IBD) pose a considerable burden on patients and society. Rehabilitation programs are increasingly being considered as a complementary treatment. However, empirical evidence on the effectiveness of this approach is still sparse. We therefore investigated changes in patient-reported outcomes and an objective biomarker throughout a three-week inpatient gastrointestinal rehabilitation.

**Methods:**

We conducted a longitudinal prospective observational study with a pretest-posttest design. Patients completed questionnaires on their subjective health-related quality of life (HRQoL), psychological distress, disease-specific burden, and work ability. Stool samples were collected to determine fecal calprotectin levels. Mean level changes were analyzed by means of *t* tests for repeated measurements, while differential changes between Crohn’s disease and ulcerative colitis patients were compared using analysis of variance.

**Results:**

Subjective disease burden and psychological distress decreased over the course of rehabilitation, whereas HRQoL and work ability increased. Fecal calprotectin levels slightly rose and were not meaningfully related to changes in subjective perception of illness.

**Conclusions:**

We present evidence on rehabilitation as an important treatment in people with IBD in terms of subjective disease burden and various indicators of personal well-being. Furthermore, we advise against the use of fecal calprotectin as a marker for monitoring disease activity in short-term rehabilitation settings.

## Introduction

Inflammatory bowel disease (IBD) is a group of chronic intestinal inflammatory conditions. The most common forms of IBD are Crohn’s disease (CD) and ulcerative colitis (UC), which are characterised by alternating phases of remission and relapse [1,2]. Much of the aetiology of these diseases remains unclear, but appears to be multifactorial and influenced by the individual’s immune system, genetic predisposition, and environmental factors [1,3]. The patient’s prognosis is unstable, with frequent changes between high disease activity and relative health [4]. The most common symptoms include diarrhoea, abdominal pain, frequent bowel movements, rapid fatigue, or weakness [5,6]. These symptoms are often accompanied by psychosocial problems that affect patients’ quality of life (QoL) and severely restrict their ability to participate in social and occupational activities [6].

In Europe, about 0.3% of the population is affected by IBD, which may appear somewhat low, yet is of considerable rehabilitative and socio-medical importance, as it poses a considerable burden on patients (i.e., a “disease burden”), as well as on healthcare systems and society at large (i.e., a “economic burden”) [7]. Although the conditions are chronic, no definitive cure is currently available and medications only target symptom relief, patients often seek supportive, complementary therapies. Therefore, rehabilitation programs for patients with IBD are increasingly being considered [1,6]. This approach seems promising, as non-pharmacological therapies (i.e., diet, exercise, and psychological treatments) have a positive impact on QoL, inflammation, and mental health in patients with IBD, especially when embedded in a holistic program, as opposed to single interventions [8].

Rehabilitation measures based on the bio-psychosocial model of the International Classification of Functioning, Disability and Health (ICF) take a holistic approach to support the restoration of patients’ participation in social and occupational activities. Rehabilitation programs for individuals with IBD that combine physical therapy, structured exercise therapy, nutritional counselling, and mental health support could optimize medical outcomes, patients’ psychophysical functioning, work capacity, and health-related quality of life (HRQoL) [9]. Indeed, inpatient rehabilitation for patients with IBD has been shown to have positive effects on social participation, disease activity, HRQoL, and self-management, compared to standard care [10]. Further evidence on rehabilitation in patients with IBD and its effectiveness is still sparse [9].

In Austria, individuals have the opportunity of applying for rehabilitation following medical treatment with the aim of either maintaining the success of the treatment or alleviating the consequences of the disease. Typically, the duration of these rehabilitation programs is three weeks, and they are conducted in either inpatient or outpatient rehabilitation centres. Individuals diagnosed with IBD, due to the chronic nature of the condition, are eligible to make regular applications for rehabilitation procedures.

Given the comprehensive scope of rehabilitation measures, it is essential to assess various outcomes throughout a rehabilitation program. Biomarkers, such as fecal calprotectin, are used to measure changes in disease progression objectively. Fecal calprotectin appears to be a suitable biomarker for monitoring inflammation in IBD patients [11]. It is a calcium-binding protein, primarily derived from neutrophils, plays a regulatory role in inflammatory processes, and has shown to be stable and resistant to bacterial degradation in feces [11]. Therefore, fecal calprotectin may potentially be a useful marker for tracking changes in disease activity during short-term settings, like rehabilitation, as well.

In addition to biomarkers, patient-reported outcomes (PROs) provide valuable insights into patients’ HRQoL, ability to work, and disease burden, providing a complementary subjective perspective. These aspects elude objective measurement, and it is, therefore, important to consider them in order to gain a comprehensive understanding of the patients’ progress [12].

Given the lack of evidence on the effectiveness of rehabilitative measures in reducing disease activity and burden, we aimed to assess changes in an objective biomarker (fecal calprotectin) and selected PROs over the course of a three-week inpatient gastrointestinal rehabilitation. We expected a reduction in calprotectin levels, subjective disease burden, and psychological distress over the course of treatment, as well as an increase in HRQoL and subjective work ability.

## Materials and methods

### Study design

The present study was conducted as a longitudinal prospective observational study with two measurement points. Prior to the collection of any data, we conducted an a-priori power analysis to derive an estimate of the required sample size. Taking into account a drop-out rate of 5% and an expected medium-sized effect, it was planned to include 200 patients (100 patients per IBD-type, i.e., CD and UC).

Patients with IBD who were admitted to the Austrian rehabilitation center in Bad Aussee for a three-week gastrointestinal rehabilitation were approached by a clinician and asked to participate in the study. Patients were given information about the study and following a consultation with a physician agreed to participate by signing a consent form. Participation was voluntary and could be withdrawn at any time during the study. All individuals aged 18 years or older with a diagnosis of either K50 (CD) or K51 (UC) according to the International Classification of Diseases (ICD) were considered eligible for inclusion. Premature termination of the rehabilitation stay resulted in exclusion from the study. Patient recruitment and data collection was conducted between August 2022 and July 2023.

### Intervention

Patients underwent a three-week inpatient gastrointestinal multidisciplinary rehabilitation program at the rehabilitation center in Bad Aussee (Austria). This multidisciplinary program is a coordinated pool of treatments and therapeutic interventions to provide the best possible physical, mental and social condition for alleviating the disease burdens in patients with IBD. The goal of the rehabilitation program may be individual for each patient, but can be generalized as maintaining or regaining optimal functioning in his or her community through their own efforts and slowing or reversing disease progression through improved health behaviors. The fundamentals of the rehabilitation program entailed interdisciplinary and problem-orientated care within the framework of the bio-psychosocial model of the International Classification of Functioning, Disability and Health (ICF). Accordingly, multidisciplinary rehabilitation is a complex and comprehensive intervention, which includes exercise training, physical therapy, patient education, psychosocial management and behavioral modification programs to improve physical, psychosocial and emotional health [13]. In Austria, a specific amount of „therapy minutes“is allocated for each rehabilitation patient, with the objective of customizing an individualized program. Patients diagnosed with IBD have 2400 minutes of therapy and treatment at their disposal. The number and type of different therapy sessions are contingent upon the degree of each patient’s impairment, as determined by the ICF framework [14]. The 2400 minutes of therapy were distributed among active therapy, passive treatments and educational measures (detailed description in Table 1). Treatment and therapeutic interventions encompass both group-based sessions, such as group exercise training, and individualized therapies, including, e.g., one-on-one physical therapy.

**Table 1:** Description of the treatment pool in multidisciplinary rehabilitation.

**Active therapy**	**Endurance exercise**	2–4 times/week for 25 min per session Supervised continuous cycle or treadmill endurance training (group-based setting)
	**Strength/ resistance exercise**	2–4 times/week for 25 min per session Supervised strength training to train legs, arms, back and core (group-based setting)
	**Physical therapy**	Individualized therapy in an One-on-One Setting (frequency depending on degree of restriction (ICF-based)) were provided (25 min per session)
	**Occupational therapy**	Individualized therapy (frequency depending on degree of restriction (ICF-based)) were provided (25 min per session) (group-based and/or One-on-One setting)
	**Physical activity**	2 times/week for 25–50 min depending on state of health and diagnosis Supervised hiking and walking exercise (group-based setting)
	**Relaxation techniques**	2 times/week for 25 min (e.g., Jacobson technique) (group-based setting)
	**Psychological counselling**	Psychological counselling and gut directed hypnosis (frequency depending on degree of restriction (ICF-based)) were provided (One-on-One setting)
	**Nutritional counselling**	Body composition and nutritional counselling (e.g., supplementary nutrition, problems with gut microbiome, etc.) were provided (frequency depending on degree of restriction (ICF-based)) (One-on-One setting)
	**(CED-) Nurse counselling**	Individual counselling (e.g., stoma, incontinency) (One-on-One setting)
**Passive therapy**		If necessary, scar treatment, massage, acupuncture, ultrasound, electrical stimulation etc. were provided (One-on-One setting)
**Patient education**		Disease and lifestyle related topics like pain consulting, CED specific consultation, career advice, nicotine advice, etc. (group-based setting)

### Outcome measures

#### Fecal calprotectin.

Stool samples were collected from patients at the time of admission and at the time of discharge. Patients were provided with illustrated instructions to ensure that stool samples were collected correctly. Each test tube was filled on average with 5 g of stool and sent to the in-house laboratory on the same day. Calprotectin remains stable in stool at room temperature for three days and in the refrigerator for up to seven days. In the laboratory, samples were stored at 5°C in a refrigerator and analyzed within one to a maximum of two days. For each patient, one to two stool samples (upon admission and discharge) were tested using an EU-Fast Reader (Eurospital, Italy).

Firstly, 56 mg of stool were mixed with 2.8 ml of buffer (salinity and pH-controlled solution) in a high-speed homogenizer. The homogenate was then centrifuged at 3,000 revolutions per minute for 10 minutes using a Rotina 35 centrifuge (Hettich Zentrifugen, Germany), before the supernatant was collected. Lastly, calprotectin levels were measured using an immunoassay. The lower limit of detection of the analytical method was 50.00 μg/g, and all observations with a value below this threshold were fixed at 50.00 μg/g.

#### EuroQol 5 dimensions 5 level version (EQ-5D-5L).

The EuroQol 5 Dimensions 5 Level Version (EQ-5D-5L) is a self-report questionnaire designed to assess subjective HRQoL. This is subdivided into five dimensions using the questionnaire, each of which is covered by a single item: (1) mobility, (2) self-care, (3) usual activities, (4) pain/discomfort, and (5) anxiety/depression. These items are rated on a five-point scale, ranging from 1 (no problems) to 5 (extreme problems). Respondents’ answers to each item can then be used to construct a health profile. Calculating a score from this health profile is also possible based on country-specific weighting factors. As no value set exists for the Austrian population, we opted to use the German value set to calculate the EQ-5D-5L score, which ranges from −0.661 (extreme problems in all dimensions) to 1 (no problems in any dimension) [15]. In addition to the aforementioned items, the EQ-5D-5L includes a visual analog scale (VAS) on which respondents are asked to assess their subjective health state on a scale from 0 (“worst health you can imagine”) to 100 (“best health you can imagine”).

#### Work ability index (WAI).

The Work Ability Index (WAI) is a self-report questionnaire that provides a subjective assessment of work ability. The questionnaire can be divided into seven dimensions:

(1) current work ability compared to the best work ability ever achieved, (2) current work ability in relation to the physical and mental demands of the job, (3) current number of medically diagnosed diseases, (4) extent of work limitations due to illnesses/injuries, (5) sick leave days during the last 12 months, (6) self-assessment of work ability in the next 2 years, and (7) mental resources and well-being. The WAI score can range from 7 to 49 points, with higher values indicating higher work ability [16].

#### Patient health questionnaire-4 (PHQ-4).

The Patient Health Questionnaire 4 (PHQ-4) is an ultra-brief screening tool designed to assess psychological distress related to anxiety and depression [17]. The questionnaire comprises a total of four items that are self-reported on a four-point scale ranging from 0 (not at all) to 3 (nearly every day). Scores are calculated by summing the individual responses. The total score can range from 0 to 12, with higher values indicating greater psychological distress [18].

#### IBD disk.

The IBD Disk is a 10-item self-report tool used to assess patients’ disease burden related to abdominal pain, bowel control, interpersonal interactions, education and work, sleep, energy, emotions, body image, sexual function, and joint pain [19]. Each item is answered on an 11-point scale, ranging from 0 (absolutely disagree) to 10 (absolutely agree). The score is calculated by summing the individual items, the result ranging from 0 to 100. Higher values indicate greater subjective disease burden.

### Statistical analyses

All statistical analyses were performed in SAS Viya V.03.05 (SAS Institute Inc.). Prior to conducting the analyses, a thorough examination was performed to determine whether all focal study variables met the requirement of normality and identify any potential outliers.

Immediate changes (admission vs. discharge) in fecal calprotectin levels, HRQoL, subjective work ability, mental impairment, and disease-specific burden in both CD and UC patients were examined separately using welch *t*-tests for paired samples, and Cohen’s *d* as the measure of effect size. The extent of differences in the immediate changes in the aforementioned parameters following rehabilitation were statistically compared between the two types of inflammatory bowel disease (CD vs. UC) using analysis of variance and by comparing the magnitude of raw and standardized mean changes. In this case, the bias-adjusted Hedges’ *g* was used as an indicator of effect size strength, as the group sizes were not identical.

We based our interpretation of findings predominantly on the strength of effect sizes and their precision rather than nominal null-hypothesis significance testing, as is strongly recommended in the current literature [20]. Nevertheless, results of null-hypothesis significance tests are reported throughout. In the case of multiple comparisons, Bonferroni-Holm adjusted p-values are reported. In terms of interpreting effect meaningfulness, we used the well-established classification of Cohen [21], with absolute values exceeding *d* and *g* = 0.20, 0.50, and 0.80, and *r* = 0.10, 0.30, and 0.50 indicating small, medium, and large effects, respectively.

In the case of missing data in individual outcomes, affected cases were excluded for these specific outcomes. The fact that premature termination of rehabilitation led to exclusion from the study meant that missing data relating to entire measurement points did not have to be addressed.

### Ethical considerations

This study was approved by the ethics committee of the Medical University of Graz (1198–2022). The participation in the study requires a signed consent form and could be terminated at any time. Refusal to participate or premature withdrawal had no adverse effects on the medical care provided to the patients.

## Results

### Patient characteristics

In all, 226 patients were recruited at the beginning of their gastrointestinal rehabilitation. A total of 13 patients prematurely terminated their rehabilitation program, resulting in a total sample of 213 patients eligible for inclusion in the study. Table 2 shows demographic and clinical characteristics of the study population at baseline (i.e., at the time of admission).

**Table 2:** Demographic and clinical characteristics of the study population at baseline.

Characteristics	N_total_	Total	N_CD_	CD	N_UC_	UC
Gender (female), N (%)	213	108 (50.7)	115	57 (49.6)	98	51 (52.0)
Age in years, mean (SD)	213	46.9 (12.3)	115	46.3 (12.3)	98	47.5 (12.3)
Disease duration in years, mean (SD)	209	13.2 (10.9)	115	14.0 (10.9)	94	12.1 (11.0)
Symptomatic (yes), N (%)	212	146 (68.9)	115	80 (69.6)	97	66 (68.0)
Surgical treatment (yes), N (%)	213	91 (42.7)	115	71 (61.7)	98	20 (20.4)
Drug treatment (yes), N (%)	213	170 (79.8)	115	92 (80.0)	98	78 (79.6)
Smoking status (yes), N (%)	213	48 (22.5)	115	38 (33.0)	98	10 (10.2)
Weight in kg, mean (SD)	213	75.4 (17.4)	115	76.1 (18.3)	98	74.5 (16.2)

Abbreviations: N_total_, total sample size; N_CD_, sample size Crohn’s disease; N_UC_, sample size ulcerative colitis; CD, Crohn’s disease; UC, Ulcerative colitis; N, sample size; SD, standard deviation.

### Overall changes in fecal calprotectin and patient-reported outcome measures

Mean changes in focal study variables (admission vs. discharge) are reported in Table 3. The data were found to be approximately normal distributed. In order to verify the robustness of the analytical process, all analyzed parameters were also subjected to non-parametric testing (e.g., Wilcoxon signed-ranks test, etc.). The findings from the non-parametric tests exhibited congruence with those derived from the parametric tests (see S1 Table in the supporting information), thereby substantiating the validity of the latter. Consequently, the more powerful parametric tests were used for the final analysis.

**Table 3:** Mean changes in focal study variables.

	N	Admission		Discharge		*t* (*df*)	*P*	*P*_adj_	Mean change [95% CI]	
		x̅	SD	x̅	SD				Unstandardized	Cohen’s *d*
Total										
Fecal calprotectin	206	187.16	217.07	212.63	241.66	1.72 (205)	.087	.261	25.48 [−3.73, 54.68]	0.11 [−0.02, 0.24]
IBD Disk	207	41.41	18.49	36.15	20.63	−4.15 (206)	<.001	<.001	−5.26 [−7.76, −2.77]	−0.27 [−0.40, −0.14]
PHQ-4	211	2.57	2.86	1.56	2.33	−8.29 (210)	<.001	<.001	−1.01 [−1.26, −0.77]	−0.39 [−0.49, −0.29]
EQ-5D-5L	209	0.86	0.18	0.90	0.16	3.76 (208)	<.001	.002	0.03 [0.02, 0.05]	0.20 [0.10, 0.30]
VAS	212	65.82	16.58	74.25	15.92	8.84 (211)	<.001	<.001	8.43 [6.55, 10.31]	0.52 [0.39, 0.64]
WAI	182	30.47	7.96	32.18	7.98	6.57 (181)	<.001	<.001	1.71 [1.19, 2.22]	0.21 [0.15, 0.28]
Crohn’s disease										
Fecal calprotectin	111	202.03	220.82	209.66	231.01	0.40 (110)	.692	.692	7.63 [−30.43, 45.70]	0.03 [−0.13, 0.20]
IBD Disk	114	42.52	18.97	37.82	21.41	−2.53 (113)	.013	.065	−4.71 [−8.40, −1.01]	−0.23 [−0.42, −0.05]
PHQ-4	113	2.63	2.85	1.66	2.47	−5.50 (112)	<.001	<.001	−0.96 [−1.31, −0.62]	−0.36 [−0.50, −0.23]
EQ-5D-5L	111	0.85	0.19	0.90	0.15	3.51 (110)	.001	.008	0.05 [0.02, 0.08]	0.29 [0.13, 0.45]
VAS	114	64.11	17.33	72.90	17.85	6.35 (113)	<.001	<.001	8.79 [6.05, 11.53]	0.50 [0.33, 0.67]
WAI	98	29.65	8.03	31.66	8.15	6.02 (97)	<.001	<.001	2.01 [1.34, 2.67]	0.25 [0.16, 0.34]
Ulcerative colitis										
Fecal calprotectin	95	169.79	212.46	216.11	254.74	2.02 (94)	.046	.184	46.32 [0.83, 91.81]	0.20 [0.01, 0.39]
IBD Disk	93	40.05	17.88	34.10	19.57	−3.58 (92)	.001	.008	−5.95 [−9.25, −2.65]	−0.32 [−0.50, −0.14]
PHQ-4	98	2.51	2.87	1.44	2.18	−6.33 (97)	<.001	<.001	−1.07 [−1.41, −0.74]	−0.42 [−0.56, −0.29]
EQ-5D-5L	98	0.88	0.17	0.89	0.17	1.54 (97)	.126	.261	0.02 [−0.00, 0.04]	0.10 [−0.03, 0.22]
VAS	98	67.80	15.52	75.81	13.24	6.18 (97)	<.001	<.001	8.01 [5.44, 10.58]	0.56 [0.36, 0.75]
WAI	84	31.43	7.82	32.79	7.77	3.34 (83)	.001	.008	1.36 [0.55, 2.17]	0.17 [0.07, 0.28]

Abbreviations: N, sample size; x̅, mean; SD, standard deviation; t, t-value; df, degrees of freedom; P, P-value; Padj, Bonferroni-Holm corrected P-values [22]; CI, confidence interval.

Fecal calprotectin levels increased over the course of the rehabilitation stay, but not statistically significant. IBD-related disease burden only decreased by a small effect but only in UC (t (92) = −3.58, *P*_adj =_.008, d = − 0.32 [−0.50, −0.14]). In both groups psychological distress decreased by small effects between the time of admission and discharge, as indicated by lower PHQ-4 values, at the end of treatment. In the total sample, the most meaningful changes regarding IBD-related disease burden were observed in the energy (*t*(206) = −9.68, *P*_adj_ < .001, *d* = −0.72 [−0.88, −0.56]), emotions (*t*(206) = −4.99, *P*_adj_ < .001, *d* = −0.38 [−0.53, −0.22]), and join*t* pain subscales (*t*(206) = −2.76, *P*_adj_ = .05, *d* = −0.17 [−0.30, −0.05]). In CD patien*t*s, non-trivial changes were related to the energy (*t*(113) = −7.08, *P*_adj_ < .001, *d* = −0.70 [−0.91, −0.49]), emotions (*t*(113) = −3.27, *P*_adj_ = .013, *d* = −0.32 [−0.52, −0.13]), and joint pain subscales (*t*(113) = −2.34, *P*_adj_ = .17, *d* = −0.20 [−0.37, −0.03]). Somewha*t* deviating from *t*his pattern, in UC patients, the biggest changes were observed in the energy (*t*(93) = −6.57, *P*_adj_ = < .001, *d* = −0.74 [−0.98, −0.49]), emotions (*t*(93) = −3.79, *P*_adj_ = .003, *d* = −0.44 [−0.68, −0.20]), and sexual func*t*ions subscales (*t*(93) = −3.17, *P*_adj_ = .016, *d* = −0.27 [−0.44, −0.10]).

Subjective HRQoL increased during the rehabilitation stay, as indicated by increasing EQ-5D-5L scores, although the change was more pronounced in CD than in UC. Patients’ ratings of their subjective health state, as indicated by the VAS, increased by a medium-sized effect over the course of treatment in both groups. Subjective work ability increased during patients’ rehabilitation, albeit by small and trivial effects for CD and UC, respectively.

### Differential changes in outcome measures between groups

Differential changes in outcome measures between groups over the course of rehabilitation are reported in Table 4. Differences in focal outcomes between groups were predominantly trivial, as reflected in their standardized mean differences (*g* range = 0.06 to 0.19), with the exception of a small difference in EQ-5D-5L scores. However, confidence intervals were broad throughout, indicating low precision, and none of the comparisons reached nominal statistical significance, as indicated by non-significant interaction terms (i.e., interaction of group and time) in the analysis of variance models. The change over time is similar in both groups.

**Table 4:** Differences in mean changes between groups and group*time interaction.

	Crohn’s Disease			Ulcerative Colitis			Difference in mean changes		Interaction (group*time)	
	N	Mean change	SD	N	Mean change	SD	Unstandardized (95% CI)	Hedges *g* [95% CI]	*F*	*P*_adj_
Fecal calprotectin	111	7.63	202.36	95	46.32	223.30	38.69 [−19.81, 97.18]	0.18 [−0.09, 0.46]	1.63	>.999
IBD Disk	114	4.71	19.89	93	5.95	16.03	1.24 [−3.79, 6.27]	0.07 [−0.21, 0.34]	0.27	>.999
PHQ-4	113	0.96	1.87	98	1.07	1.68	0.11 [−0.39, 0.59]	0.07 [−0.21, 0.33]	0.23	>.999
EQ-5D-5L	111	0.05	0.15	98	0.02	0.10	0.03 [−0.00, 0.07]	0.23 [−0.04, 0.51]	3.16	0.462
VAS	114	8.79	14.78	98	8.01	12.83	0.78 [−3.00, 4.56]	0.06 [−0.21, 0.33]	0.18	>.999
WAI	98	2.01	3.30	84	1.36	3.72	0.65 [−0.38, 1.68]	0.19 [−0.11, 0.48]	1.49	>.999

Abbreviations: N, sample size; SD, standard deviation of mean change; CI, confidence interval; *F,* F-value; *P*_adj_, Bonferroni-Holm corrected *P*-values [22].

### Relationship between fecal calprotectin and patient-reported outcome measures

Calprotectin levels were not meaningfully related to any patient-reported outcome measures at the time of admission (*r* range −.03 to.02), with the exception of a small correlation with WAI scores (*r* = −.12, 95% CI [−.26,.03], *P* = .119). Calprotectin levels at discharge exhibited small negative correlations with the total PHQ (*r* = −.16, 95% CI [−.29, −.03], *P* = .019) and IBD-Disc scores (*r* = −.12, 95% CI [−.26,.02], *P* = .082), i.e., while calprotectin levels increased, psychological distress and patients’ disease burden decreased. All other associations were of trivial size (*r* = −.01 to.04 for EQ-5D-5L and WAI scores, respectively).

## Discussion

Here, we present compelling new evidence about the role of rehabilitation measures in alleviating subjective disease burden and psychological distress, as well as in improving subjective HRQoL and work ability in patients with IBD. Furthermore, we demonstrate and discuss that fecal calprotectin, as an objective biological parameter, does not appear to be suitable as a monitoring parameter for assessing therapeutic success in this rehabilitation setting, and it does not meaningfully reflect subjective well-being.

Over the course of rehabilitation, the subjective overall disease burden, as measured by the IBD Disk score, decreased by a small effect for CD and UC patients alike. The biggest improvement was seen in energy levels, i.e., patients reported meaningfully reduced tiredness and felt more refreshed during the day at the time of discharge than at the time of admission. Previously, the introduction of regular physical activity has been associated with improvements in physical fatigue in CD patients [23]. Furthermore, psychological treatments have shown to be promising in reducing fatigue in patients with IBD [24]. Since these specific single interventions showed some promise in targeting IBD-related fatigue, it seems plausible that a multifaceted approach may have synergetic and beneficial effects. Our results – based on a multidisciplinary rehabilitation treatment – are in line with this assumption, and may position rehabilitation measures as an effective form of complementary treatment for subjective disease burden in IBD.

Patients also reported reduced symptoms of anxiety and depression, corresponding to lower PHQ-4 and Emotion-scores in the IBD Disk. Recent evidence suggests that behavioral therapies are not only beneficial in the treatment of depression and anxiety, but may also be effective in reducing IBD-related symptoms [25]. Furthermore, previous research suggested a meaningful reduction in PHQ-4 scores following inpatient medical rehabilitation in a German IBD sample [10]. Our results are consistent with these findings, yielding comparable effect size estimates for the reduction in depressive and anxiety symptoms over the course of treatment.

Subjective HRQoL improved significantly and meaningfully over the course of treatment, with small to medium-sized effects found in the EQ-5D-5L scores and the VAS, respectively. Comparing our results with a study on the role of medical rehabilitation in IBD patients from Germany [10], we found that while in both samples, treatment was followed by increased HRQoL (based on the VAS), the improvement in our sample even exceeded that of the comparative study. It should be noted, however, that the patients’ responses differed substantially between both measures. While subjective HRQoL, as measured by the EQ-5D-5L score, improved by a small effect (excerpting UC patients, where the change was trivial in size), it increased by a medium-sized effect in the VAS. This suggests that respondents’ global view of their health state was more affected by the rehabilitation program than the changes felt in specific domains of HRQoL.

Patients showed limited work capacity at the beginning of rehabilitation. Throughout treatment, an increase in subjective work ability was observed, albeit only to a small degree. It should be noted, however, that between measurements, patients were on sick leave due to the rehabilitation treatment and, therefore, may not have been able to gauge their own work ability in a sufficiently meaningful way. Accordingly, future research should employ follow-up measurements to allow patients to return to work and subsequently assess their work capacity more accurately.

Contrary to our expectations, fecal calprotectin increased slightly over the course of rehabilitation, in both UC and CD patients, but mean values remained relatively low throughout, being indicative of mild disease activity [26]. However, possible explanations for this noteworthy finding are manifold. First, the interval between our two measurement points (i.e., three weeks of inpatient rehabilitation) could have been too short to adequately show the possible effects of the intervention in fecal calprotectin levels, as opposed to the subjectively assessed PROs. With most drug therapies, the first signs of decreased fecal calprotectin levels appeared after eight weeks and later [27]. It could be possible that multimodal interventions – like rehabilitation – also have a delayed efficacy in affecting biological markers. Second, fecal calprotectin is characterized by substantial intra-individual variability [28,29]. Due to this variability, resampling of stool samples could be used to detect these intra-individual patterns. However, this approach seems unfeasible because of the relatively short period of inpatient care in most rehabilitation settings provided in Austria. Third, fecal calprotectin levels can be affected by a multitude of factors, such as non-steroidal anti-inflammatory drug intake [29], comorbidities (e.g., gastroenteritis, gastrointestinal malignancies, reflux disease, cystic fibrosis, and diverticulitis) [30], age [31] and dietary exposure [32].

We also assessed if – and to what extent – objective indicators of intestinal inflammation (i.e., fecal calprotectin) correspond to various subjective outcomes relating to HRQoL, psychological distress, subjective disease burden, and work ability in patients with IBD. Contrary to our expectations, we found that these aspects were virtually unrelated. Even more surprising, in cases of associations, these were counterintuitive. For example, upon discharge, we found that higher inflammatory activity was linked to reduced psychological distress. Similarly, increased calprotectin levels were associated with reduced subjective disease burden, as indicated by lower IBD Disk values. While these relationships could indeed be Type-I errors, they should be subject to further investigation to disentangle a potential area of future research from mere statistical artefacts. The lack of substantial correlations between subjective and objective markers of rehabilitation success and the various factors that – independently from rehabilitative measures – influence calprotectin levels, caution against using fecal calprotectin as such. This suggests that fecal calprotectin is not a suitable biomarker for monitoring disease activity in a short-term rehabilitation setting.

### Limitations

However, this study also has some limitations. Firstly, while the sample size was adequate in terms of overall mean changes in the total sample and subgroup analyses (according to the a-priori power analyses) it appeared to be insufficient for the analyses of interaction terms in the analysis of variance models. This resulted in a lack of power, as indicated by small effects that failed to achieve nominal statistical significance. Furthermore, lack of study power often leads to the possibility of effect size estimates being inflated [33]. Consequently, our findings regarding the analyses of variance models should be interpreted with caution. Therefore, high-powered future research is needed to obtain robust estimates of the differential changes in focal study parameters between CD and UC patients.

Second, the outcomes observed here were limited to immediate changes over the course of a three-week inpatient rehabilitation program. Although these results appear promising in the short term, research on longer-term effects is needed to gain deeper insight into the role of rehabilitation in improving patients’ subjective disease burden, psychological distress, HRQoL, and work ability over a longer period.

Third, as our study was not based on a randomized intervention-control design, the observed changes over the course of rehabilitation cannot be causally attributed to the investigated intervention. These changes may be the result of natural recovery processes or other influencing factors, such as being in a structured environment away from home and personal routines. Furthermore, we did not explicitly account for further potential confounding factors such as between-subjects variation in diet, medications, exercise, and psychological interventions. Therefore, further research is needed to address the complexity of a multimodal and multidisciplinary rehabilitation for IBD-patients.

## Conclusions

Here, we provide empirical evidence suggesting that multidisciplinary medical rehabilitation is an important treatment in patients with Crohn’s disease and ulcerative colitis. During the three-week inpatient gastrointestinal rehabilitation, the subjective disease burden and psychological distress meaningfully decreased, while HRQoL and work ability improved. Additionally, although it may be valuable in long-term monitoring of intestinal inflammation, we advise caution against the use of fecal calprotectin as an indicator of rehabilitation success in short-term settings.

## Supporting information

S1 Table Results of the non-parametric analysis (Wilcoxon sign-ranked test). Abbreviations: N, sample size; IQR, Interquartile range; P, P-value; P_adj_, Bonferroni-Holm corrected P-values.
(DOC)
