# Supplementary material for: Changes in patient-reported outcomes (PROs) and fecal calprotectin levels in patients with inflammatory bowel disease following a three-week gastrointestinal inpatient rehabilitation
Source: PLoS One. 2025 Aug 29;20(8):e0330922. doi: 10.1371/journal.pone.0330922 (PMC12396678; doi:10.1371/journal.pone.0330922)
Supplement: S1 Table — Abbreviations: N, sample size; IQR, Interquartile range; P, P-value; Padj, Bonferroni-Holm corrected P-values. (DOC) [file pone.0330922.s001.doc]

|  |  | Admission | | Discharge | |  |  |  | Mean median change [IQR] |
| --- | --- | --- | --- | --- | --- | --- | --- | --- | --- |
|  | N | Median | IQR | Median | IQR | Statistics [S] | *P* | *P*_adj_ | Unstandardized |
| Total |  |  |  |  |  |  |  |  |  |
| Fecal calprotectin | 206 | 77.35 | 202.5 | 112.05 | 214.6 | 1305 | 0.035 | .070 | 0.0 [-26.6 – 84.5] |
| IBD Disk | 207 | 40 | 27 | 35 | 30 | 3783 | < .001 | .002 | 6.0 [-3.0 – 15.0] |
| PHQ-4 | 211 | 2 | 4 | 1 | 3 | 2783 | < .001 | .002 | 0.0 [0.0 – 2.0] |
| EQ-5D-5L | 209 | 0.91 | 0.12 | 0.94 | 0.12 | 1615 | .001 | .008 | 0.0 [0.0 – 0.05] |
| VAS | 212 | 70 | 25 | 80 | 15 | 5272.5 | < .001 | .002 | 10 [0.0 – 15.0] |
| WAI | 182 | 31 | 11 | 35 | 11 | 3453 | < .001 | .002 | 1.3 [0.0 – 4.0] |
| Crohn’s disease |  |  |  |  |  |  |  |  |  |
| Fecal calprotectin | 111 | 98.6 | 220 | 112.1 | 230.3 | 70 | .784 | .784 | 0.0 [-38.6 – 60.7] |
| IBD Disk | 114 | 45 | 28 | 36 | 30 | 943 | .003 | .015 | 5.5 [-5.0 – 15.0] |
| PHQ-4 | 113 | 2 | 4 | 1 | 3 | 792.5 | < .001 | .002 | 0.0 [0.0 – 1.0] |
| EQ-5D-5L | 111 | 0.913 | 0.162 | 0.943 | 0.132 | 508.5 | .001 | .008 | 0.0 [0.0 – 0.5] |
| VAS | 114 | 65 | 25 | 80 | 20 | 1433.5 | < .001 | .002 | 9.0 [0.0 – 19.0] |
| WAI | 98 | 31 | 12 | 34.75 | 10 | 1254 | < .001 | .002 | 1.5 [0.0 – 4.0] |
| Ulcerative colitis |  |  |  |  |  |  |  |  |  |
| Fecal calprotectin | 95 | 62 | 171.9 | 108.22 | 214.6 | 519.5 | .005 | .020 | 6.7 [-6.9 – 108.9] |
| IBD Disk | 93 | 38 | 27 | 31 | 30 | 943.5 | < .001 | .002 | 6.0 [-2.0 – 16.0] |
| PHQ-4 | 98 | 2 | 4 | 1 | 2 | 613 | < .001 | .002 | 0.0 [0.0 – 2.0] |
| EQ-5D-5L | 98 | 0.913 | 0.119 | 0.943 | 0.123 | 316 | .009 | .027 | [0.0 – 0.05] |
| VAS | 98 | 70 | 20 | 80 | 15 | 1237.5 | < .001 | .002 | 10.0 [0.0 – 15.0] |
| WAI | 84 | 32.25 | 11 | 35 | 11 | 557.5 | .001 | .008 | 1.0 [-1.0 – 3.5] |
| **S1 Table.** Results of the non-parametric analysis (Wilcoxon sign-ranked test). Abbreviations: N, sample size; IQR, Interquartile range; P, P-value; P_adj_, Bonferroni-Holm corrected P-values. | | | | | | | | | |
